# Quantitative Study of Physical, Social, Psychological, and Environmental Challenges Faced by Patients With Drug-Resistant Tuberculosis

**DOI:** 10.7759/cureus.69694

**Published:** 2024-09-19

**Authors:** Tushar Sahasrabudhe, Mithun Nilgiri K

**Affiliations:** 1 Respiratory Medicine, Dr. D. Y. Patil Medical College, Hospital & Research Centre, Dr. D. Y. Patil Vidyapeeth, Pune, IND

**Keywords:** drug-resistant tuberculosis (dr-tb), mental health, national tuberculosis elimination program (ntep), psychometric assessment, public health, quality of life (qol), social interactions, socioeconomic challenges, whoqol-bref

## Abstract

Background

This study chronicles the socioeconomic and emotional challenges experienced by patients suffering from drug-resistant tuberculosis (DR-TB) in India, the country with the highest tuberculosis (TB) burden globally. Current government measures under the National TB Elimination Programme (NTEP) that include widely available molecular diagnostic methods, well-defined DR-TB regimens, free drug distribution, treatment adherence strategies, etc. need to expand to cover socioeconomic and emotional aspects of the disease.

Objective

The objective of this study was to assess the effects of DR-TB and its treatment on the quality of life of patients in the psychological, social, environmental, and physical domains using validated scales.

Method

Conducted at a tertiary care center in Pune, this quantitative study utilized the World Health Organization Quality of Life (WHOQOL-BREF) questionnaire to objectively assess the quality of life of 64 DR-TB patients. The questionnaire was self-administered but assisted by the investigator to understand the meaning of the questions, and it covered four domains: psychological, social, environmental, and physical.

Results

The results indicated significant psychological distress, with the psychological domain scoring the lowest mean (33.41), followed by social (35.52), environmental (41.27), and physical domains (41.88). These findings underscore the profound impact of DR-TB on the mental health, social interactions, and overall well-being of these patients. Furthermore, substantial employment challenges, financial constraints, and fears of disease transmission were prevalent among patients, highlighting socioeconomic disparities.

Conclusion

The study emphasizes the necessity of holistic interventions that include psychological support, socioeconomic empowerment, and public health campaigns to improve the quality of life and treatment adherence for DR-TB patients. Future research should explore integrated care models that address both the clinical and psychosocial needs of patients. The insights from this study suggest a need for policy enhancements and resource allocation to better support this vulnerable population, ultimately aiming for more comprehensive and sustainable DR-TB management, thus focusing not only on the outcome but also on morbidity.

## Introduction

India accounted for the highest number of tuberculosis (TB) cases globally in 2022, with 2.8 million TB cases, representing 27% of the global burden (WHO Global TB Report 2023) [1]. The total global TB burden included an estimated 10.6 million new TB cases, a 4.5% rise from 2021, and around 1.6 million TB-related deaths, maintaining its status as one of the leading infectious disease killers worldwide. India, Indonesia, China, the Philippines, and Pakistan together represented nearly 60% of the global TB burden.

Despite advancements in TB control, India faces significant challenges, such as drug-resistant TB (DR-TB) and the need for better healthcare infrastructure and access. Multidrug-resistant TB (MDR-TB) and extensively drug-resistant TB (XDR-TB) continue to be major issues, with approximately 450,000 new MDR-TB cases reported globally in 2022, over half of which were in India, China, and the Russian Federation [1-3].

This leaves communities vulnerable to contracting DR-TB strains that are spread via the air, particularly in high-density communities and among people living with HIV/AIDS [1,4].

The development of DR-TB is usually attributable to inadequate patient management and a lack of adherence to the approved first-line treatment regimen [5,6]. Despite the availability of effective second-line drugs to treat DR-TB and well-defined DR-TB regimens offered by various national programs, the percentage of DR-TB cures can be achieved in less than 50% of cases worldwide. The primary reasons for such poor outcomes are the extended period of treatment (which typically lasts 18 months) and the toxicity of the drugs [7], both of which lead to a lack of adherence to treatment. Moreover, psychosocial concerns frequently affect treatment adherence [8].

There has been an increase in the prevalence of DR-TB due to indiscriminate prescribing practices [9,10] among private practitioners and the inability of patients to finance the entire course of therapy provided by private physicians [11]. There are effective positive steps taken by the Indian government to address these issues by implementing well-defined regimens, free distribution of drugs, regular follow-up of the patients for ensuring treatment adherence and sputum conversion, providing flexibility in treatment modification for selective drug resistance diagnosed later on, culture or molecular methods, and guidelines for managing adverse drug effects. It has also ensured compulsory TB notification using the Ni-kshay platform, under the purview of the National TB Elimination Programme (NTEP) [12].

Research on the psychosocial context of DR-TB is scarce and poorly understood, despite advancements in research on the microbiological and clinical aspects of DR-TB. The purpose of this study was to examine the psychosocial obstacles faced by DR-TB patients, which in turn have a significant impact on treatment adherence and outcome, using objectively validated scales. This is believed to be a critical unmet need in society, as it significantly affects treatment outcomes and patient’s quality of life. This study attempts to fill this gap by providing a comprehensive understanding using objective scales.

## Materials and methods

The aim of the study was to objectively understand the quality of life (QoL) of patients suffering from DR-TB using a validated World Health Organization (WHO) scale and questionnaire.

This was a quantitative study done at a tertiary care center in Pune, which also has a DR-TB center approved by the NTEP. All patients attending outpatient clinics or admitted to the wards of the respiratory medicine department, diagnosed with DR-TB, and on treatment for a minimum of six months were considered eligible to enroll in the study. Patients were also shortlisted from Ni-kshay records and invited to participate in the study.

The inclusion criteria were age greater than 18 years, willingness to provide written informed consent, microbiological evidence of DR-TB, and completion of at least six months of DR-TB treatment (out of 18 months of an all-over longer regimen or 12 months of a shorter regimen as defined by the NTEP), as this was considered a sufficient time interval to assess the objectives.

Those not willing for a detailed interview, insufficient documentation to support the patient's story, and lack of language proficiency were excluded.

In a similar study, it was reported that MDR-TB imposes a significant economic burden on patients due to its lengthy and complex treatment regimen [13]. Socioeconomic barriers play a major role, with 23% of MDR-TB patients discontinuing treatment due to financial challenges [13]. Using WinPepi version 11.38, the minimum sample size calculated with a 95% confidence interval and an allowable error of 15 per 100 was 31.

After obtaining written and informed consent, a suitable date, time, and venue convenient for both the patient and the investigator was scheduled. Patients were accompanied by a relative involved in their care. All investigation reports and treatment records were viewed, and all the necessary data were extracted to confirm the inclusion or exclusion. The patient was asked to fill out the questionnaire for quantitative assessment, which was assisted by the investigation team and the relative for better clarity.

The WHO defines QoL as an individual’s perceptions of his position in life within the context of his culture and value systems, relative to his goals, expectations, standards, and concerns. To measure QoL across different cultures, the World Health Organization Quality of Life (WHOQOL) Group, in partnership with 15 international field centers, developed the WHOQOL assessment tool.

The WHOQOL-BREF is a condensed version of the WHOQOL-100, created using data from the field-trial version of the latter [14]. This shorter 26-item tool aligns with the WHO’s definition of QoL and is suitable for both research and clinical purposes. It is designed for situations where time is limited or to reduce the burden on respondents, making it ideal for large-scale epidemiological studies and clinical trials. Despite its brevity, the WHOQOL-BREF captures comprehensive information about various aspects of life and is easy to administer. It is available in 19 languages and typically assesses QoL over the past two weeks, although different time frames may be necessary for specific applications.

The WHOQOL-BREF is a self-administered questionnaire that evaluates four domains of QoL: physical health (seven items), psychological health (six items), social relationships (three items), and environment (eight items). It also includes two items that assess the overall QoL and general health. Responses are rated on a five-point Likert scale, with scores converted to a 0-100 scale for comparison with the WHOQOL-100. While self-administration is preferred, interviewer assistance is allowed if needed. The WHOQOL-BREF is organized into the following domains and items: (1) physical health (raw score range: 7-35); (2) psychological health (raw score range: 6-30); (3) social relationships (raw score range: 3-15); (4) environment (raw score range: 8-40).

The facets incorporated within these domains have been tabulated in Table 1.

**Table 1:** Various facets incorporated within domains.

Domain	Facets incorporated within domains
1. Physical health	Activities of daily living
	Dependence on medicinal substances and medical aids
	Energy and fatigue
	Mobility
	Pain and discomfort
	Sleep and rest
	Work capacity
2. Psychological	Bodily image and appearance
	Negative feelings
	Positive feelings
	Self-esteem
	Spirituality/religion/personal beliefs
	Thinking, learning, memory, and concentration
3. Social relationships	Personal relationships
	Social support
	Sexual activity
4. Environment	Financial resources
	Freedom, physical safety, and security
	Health and social care: accessibility and quality
	Home environment
	Opportunities for acquiring new information and skills
	Participation in and opportunities for recreation/leisure activities
	Physical environment (pollution/noise/traffic/climate)
	Transport

Statistical analysis

We extracted data variables related to the study objectives from the standard questionnaire used. The data were entered in Microsoft Excel (Microsoft Corporation, Redmond, WA) and analysis was done using SPSS 26.0 software (IBM Corp., Armonk, NY). Appropriate tests of statistical significance such as the mean and standard deviation were performed and the ANOVA test was used to compare the mean difference between the groups.

## Results

Among the 64 patients surveyed, there was a slight majority of females (51.6%) compared to males (48.4%) (Table 2).

**Table 2:** Gender distribution of patients.

Gender	Number of patients	Percentage
Female	33	51.6
Male	31	48.4
Total	64	100.0

About two-thirds of the patients came from nuclear families (62.5%), while 37.5% were from joint families (Table 3).

**Table 3:** Family type distribution among patients.

Family type	Number of patients	Percentage
Joint	24	37.5
Nuclear	40	62.5
Total	64	100.0

Of the patients, 59.4% were married, and 34.3% were single individuals, with separated and widowed patients each constituting a smaller proportion (3.1% each) (Table 4).

**Table 4:** Distribution of marital status among patients.

Marital status	Number of patients	Percentage
Married	38	59.4
Separated	2	3.1
Single	22	34.4
Widowed	2	3.1
Total	64	100.0

A large proportion of patients (82.8%) did not have any comorbid conditions, with diabetes mellitus being the exception (12.5%) (Table 5).

**Table 5:** Distribution of comorbidities among patients.

Comorbidities	Number of patients	Percentage
Coronal artery disease	1	1.6
Diabetes mellitus	8	12.5
Hypertension	1	1.6
Hypertension and diabetes mellitus	1	1.6
Nil	53	82.8
Total	64	100.0

**Figure 1:**
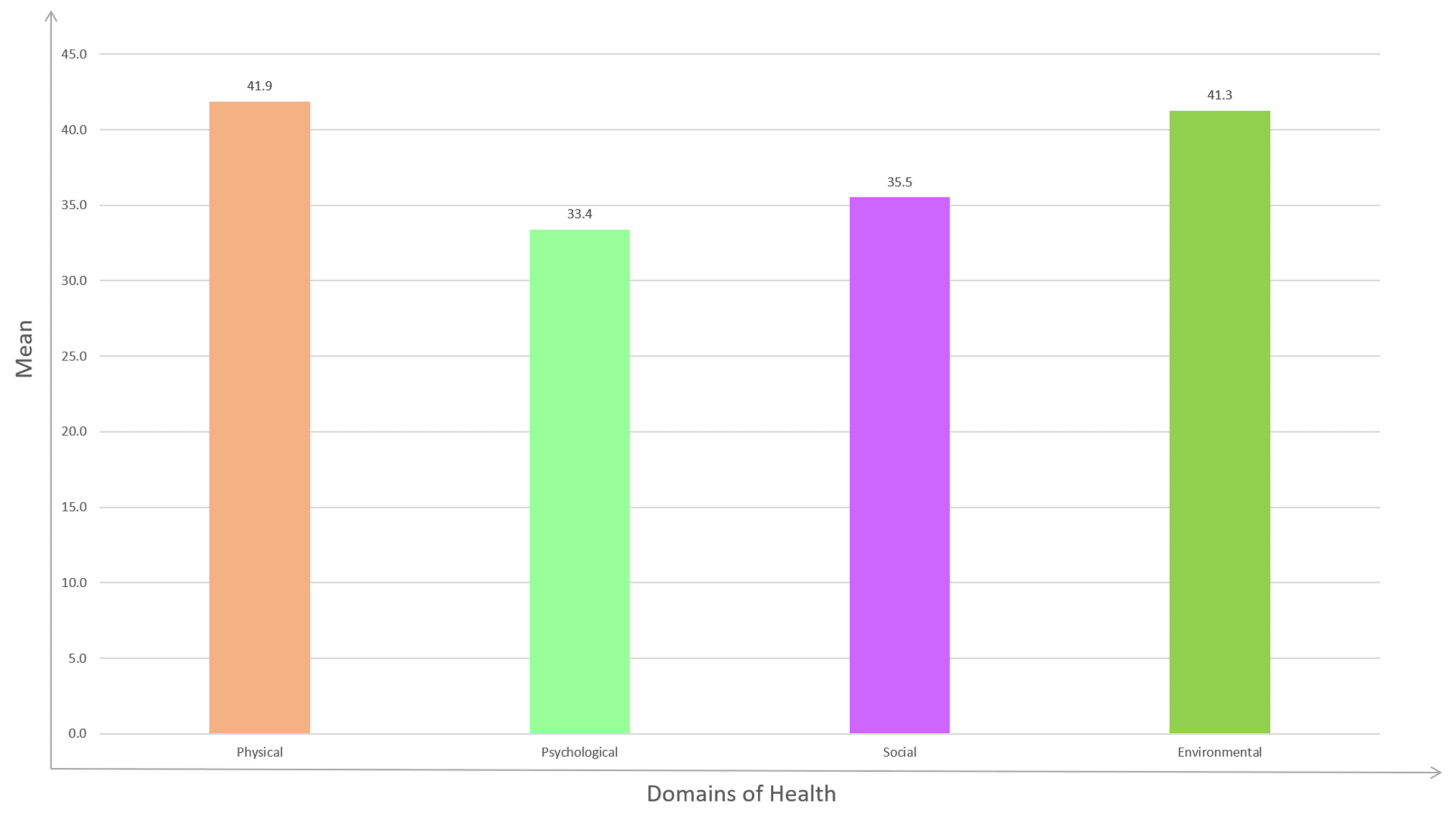
Bar diagram showing domain-wise quality of life of drug-resistant tuberculosis patients.

The majority of patients reported no substance abuse (78.125%). Nearly all patients received treatment under the NTEP regimens (98.4%), with a negligible percentage opting for private healthcare (1.6%), emphasizing the benefit of free medication and thus a great relief from the financial burden that might have been incurred due to costly medicines that were borne by the government. The findings have been summarized in Table 6 and Figure 1. Mean is directly proportional to quality of life. The higher the score, the higher the quality of the domain quality, and vice versa.

**Table 6:** Domains of quality of life (N = 64).

Domains	Minimum	Maximum	Mean ± standard deviation
Physical	6	69	41.88 ± 12.304
Psychological	0	81	33.41 ± 17.078
Social	0	94	35.52 ± 20.831
Environmental	0	88	41.27 ± 17.32

As the mean increases, domain-wise quality of life increases, and vice versa.

Physical domain

The mean score in our study population was 41.88. This domain represents how patients perceive their physical health, mobility, pain levels, and ability to perform daily activities. A higher mean suggests that, on average, patients report relatively better physical well-being despite the challenges of DR-TB. Most patients in our study mentioned that the physical component of the morbidity was not very challenging, though the drug's adverse effects were troublesome.

Environmental domain

The mean score in our study population was 41.27. This domain reflects patient’s perceptions of their living conditions, financial stability, healthcare access, and environmental support. A slightly lower mean compared to the physical domain indicates a generally positive but somewhat mixed assessment of environmental factors influencing quality of life. Most patients in our study mentioned that the environmental component of the morbidity was not very challenging, as they received good support from the NTEP.

Social domain

The mean score in our study population was 35.52. This domain evaluates aspects like social relationships, support networks, and participation in social activities. A lower mean score compared to physical and environmental domains suggests challenges in maintaining social connections and receiving adequate social support. Many patients in our study suffered significant family and social setbacks as they had to face social distancing, disturbed marital relations, and pregnancy-related concerns.

Psychological domain

The mean score in our study population was 33.41. This domain assesses psychological well-being, including self-esteem, emotional stability, spirituality, and coping abilities. The psychological domain had the lowest mean score among all domains, indicating significant challenges in psychological resilience and emotional health. This was the worst-affecting domain for our study participants. Fear of death, fear of disease transmission, anxiety, and guilt prevailed in many.

In summary, while the physical and environmental domains showed a relatively better perceived quality of life among DR-TB patients, the social and especially psychological domains require attention and targeted support to enhance overall well-being in these individuals. The psychological domain was the most affected domain among the four domains of quality of life.

**Figure 2:**
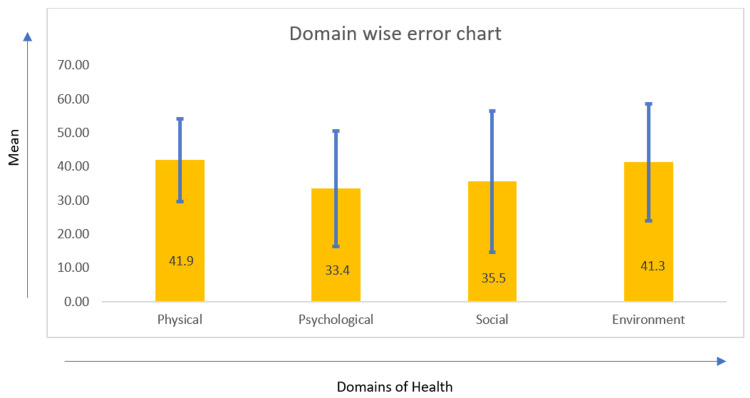
Domain wise error chart. Blue lines indicate the error chart in each domain.

On comparing the overall mean scores of physical, psychological, environmental, and social domains there is a significant mean difference between the domains with a p-value of 0.010, which is significant in all domains (Figure 2).

## Discussion

The findings of this study reveal a multifaceted burden of DR-TB, extending beyond the physical implications of the disease to significant psychological, social, and environmental challenges. The mean scores of the WHOQOL-BREF domains underscore the profound impact of DR-TB on patients' quality of life, with the psychological domain scoring the lowest (33.41), followed by the social (35.52), environmental (41.27), and physical (41.88) domains. This quantitative assessment provides critical insights into the areas requiring intervention for holistic patient care.

Psychological challenges

The psychological domain's mean score was 33.41, the lowest among all domains, which highlights the severe emotional distress faced by DR-TB patients. This distress primarily originates from prolonged treatment regimens, the stigma associated with TB, and the fear of disease transmission and relapse. Prolonged morbidity, more frequent doctor visits, and financial losses in terms of treatment costs and job opportunities add to it. Some researchers have shown that mental health issues, including depression and anxiety, are prevalent among TB patients, exacerbated by the chronic nature and societal stigma of the disease [15]. These findings signify the need for integrated mental health services within TB care programs, including regular psychological evaluations, counseling, and support groups to help patients manage stress, build resilience, and improve their emotional well-being.

Social challenges

The social domain score of 35.52 reflects considerable challenges in maintaining social as well as marital relationships and receiving adequate social as well as family support. Social isolation, resulting from stigma and the need to prevent disease transmission, can lead to loneliness and decreased social participation. Additionally, the burden on caregivers and family dynamics can strain relationships. Thomas et al. have reviewed many social challenges faced by DR-TB patients [15]. Strategies to address these issues include community-based support programs, social reintegration initiatives, and public awareness campaigns to reduce stigma. Ensuring patients have robust support networks is crucial for improving their social well-being and adherence to treatment. Creating DR-TB support groups involving successful survivors of DR-TB may help to encourage these patients and uplift their morale.

Environmental challenges

With a mean score of 41.27, the environmental domain indicates a moderate level of satisfaction with living conditions, financial stability, healthcare access, and environmental support. However, financial constraints remain a significant issue, as DR-TB treatment often leads to substantial economic burdens due to prolonged treatment periods, loss of income, and healthcare costs. Policymakers should consider enhancing financial support systems, such as direct economic aid, job placement services for patients and their families, and improving healthcare infrastructure to ensure accessible and quality care. Offering online work permission may be a great relief.

Physical challenges

The physical domain, with a mean score of 41.88, suggests that while patients report relatively better physical health compared to other domains, there are still notable challenges related to mobility, pain management, and the ability to perform daily activities. The physical toll of DR-TB and its treatment can be debilitating, emphasizing the need for comprehensive rehabilitation programs that include physical therapy, pain management, nutritional support, and continuous medical monitoring to mitigate side effects and enhance patients' physical capabilities.

Few studies have been done investigating the socioeconomic and emotional challenges faced by patients suffering from DR-TB. Sharma et al. (2013) conducted a cross-sectional study on 60 participants and found that the mean quality of life scores for MDR-TB patients were statistically significantly lower across all domains (psychological, social, and environmental) compared to patients with drug-susceptible TB, indicating a profound impact on overall well-being [16].

Clinical application

This study underscores the need for holistic interventions to tackle the multifaceted challenges of DR-TB patients by integrating psychological support, social services, and economic assistance. Multidisciplinary healthcare teams, including psychologists, social workers, and economic advisors, can provide comprehensive care. Enhancing patient education and counseling can improve treatment adherence and outcomes. Policymakers should incorporate psychosocial support into TB programs, ensuring access to mental health services, social support, and financial aid, while public health campaigns can reduce TB stigma and raise awareness. These measures can significantly improve the quality of life and treatment adherence for DR-TB patients.

Future research should explore integrated care models that address both the clinical and psychosocial needs of DR-TB patients. Longitudinal studies and examining the specific needs of subgroups, such as women, children, and those with comorbid conditions, can help tailor interventions more effectively, enhancing the overall quality of life and sustainable management of the disease.

Limitations

The study's limitations include a small sample size of 64 cases and its restriction to a single center, which may affect generalizability. Additionally, the research conducted in a tertiary care hospital introduces potential hospital-specific biases.

## Conclusions

We found it significant that DR-TB and its treatment have a significant impact on the psychological and emotional well-being of the patients, thus adding to their physical suffering. This adds to their morbidity and also affects their social and family behaviors. This study has tried to quantify the extent of the impact in an objective way.

Solid data that can be generated with an in-depth study of these domains of morbidity can then be compiled and incorporated into future versions of NTEP. Many of these issues can be addressed at the social worker level and the community level. An adequate support system can thus be created, which may include patient education, counseling, media awareness, social support, and offering psychological help.

## Figures and Tables

**Figure 3:**
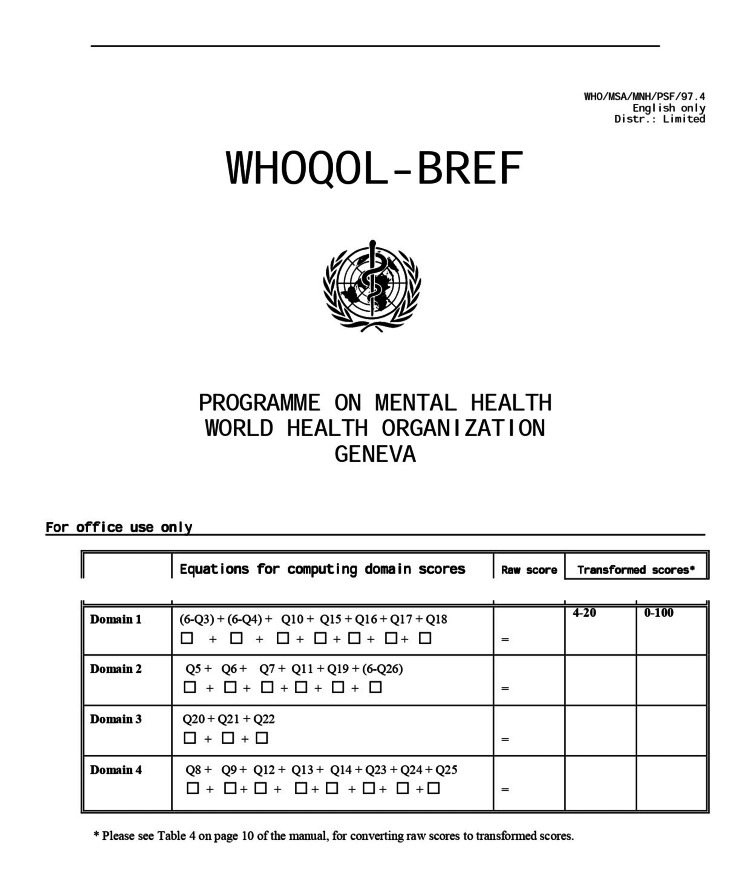
WHOQOL-BREF English version.

**Figure 4:**
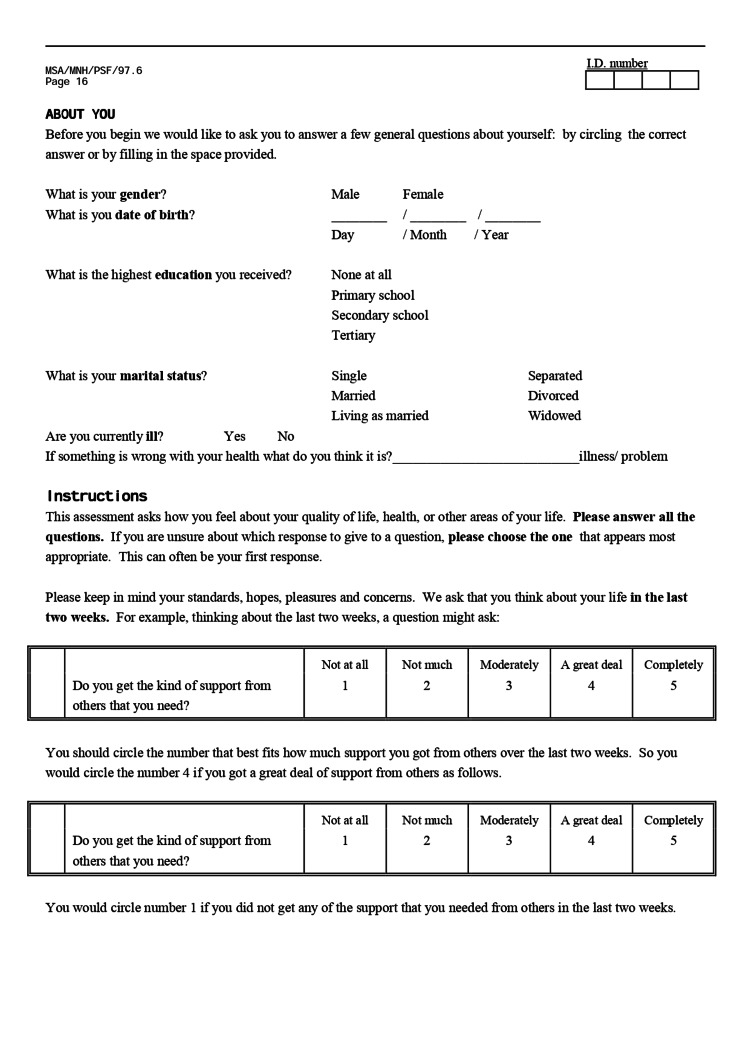
WHOQOL-BREF English version.

**Figure 5:**
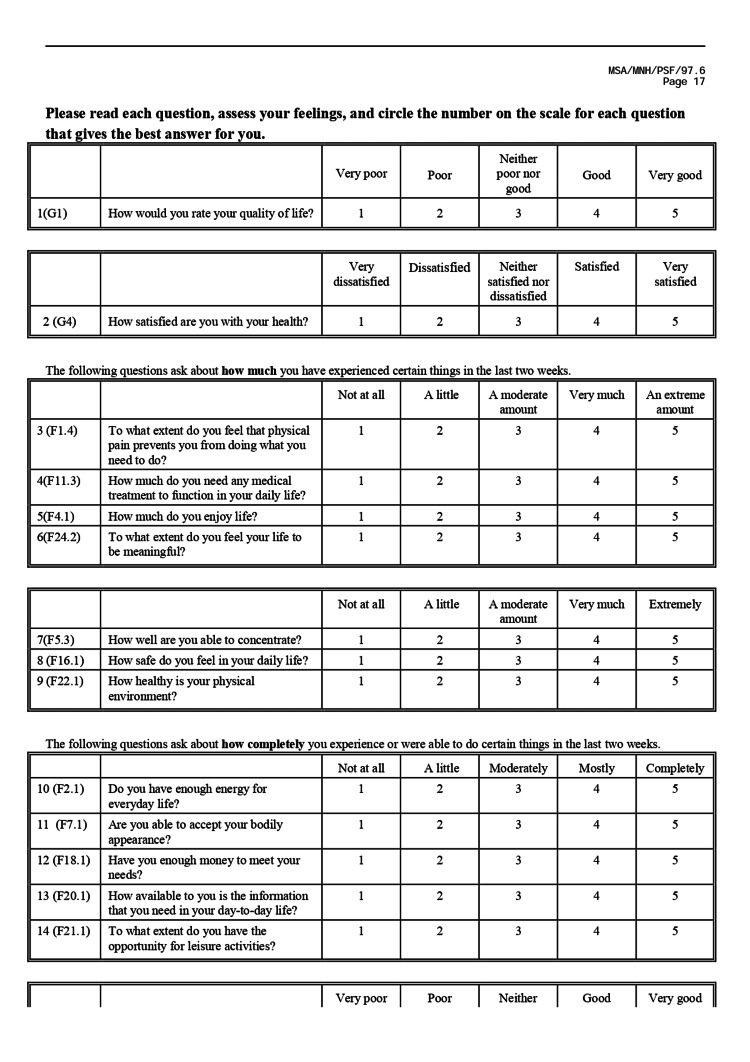
WHOQOL-BREF English version.

**Figure 6:**
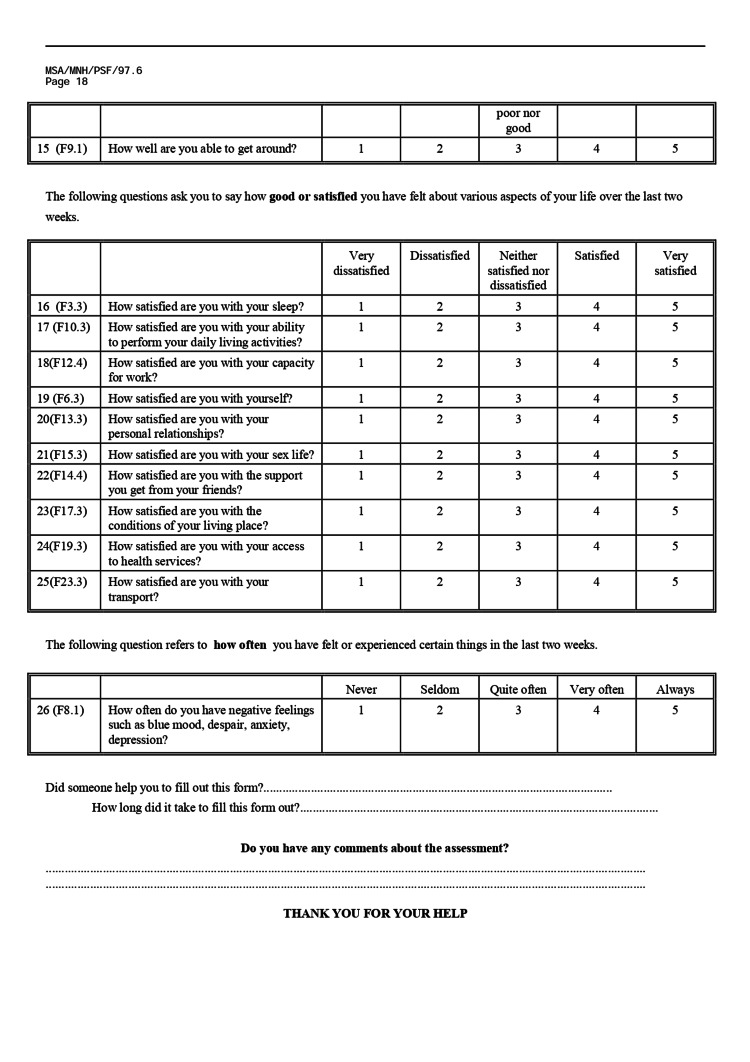
WHOQOL-BREF English version.

## Appendices

The WHOQOL-BREF questionnaire is depicted in Figures 3-6.
